# The Longitudinal Interplay between Sleep, Anthropometric Indices, Eating Behaviors, and Nutritional Aspects: A Systematic Review and Meta-Analysis

**DOI:** 10.3390/nu15143179

**Published:** 2023-07-18

**Authors:** Martina Grimaldi, Valeria Bacaro, Vincenzo Natale, Lorenzo Tonetti, Elisabetta Crocetti

**Affiliations:** Department of Psychology “Renzo Canestrari”, University of Bologna, 40126 Bologna, Italy; valeria.bacaro2@unibo.it (V.B.); vincenzo.natale@unibo.it (V.N.); lorenzo.tonetti2@unibo.it (L.T.); elisabetta.crocetti@unibo.it (E.C.)

**Keywords:** adolescents, sleep, anthropometric indices, obesity risk, eating behaviors, systematic review, longitudinal

## Abstract

Sleep is fundamental for adolescents’ healthy development but undergoes dramatic changes in quantity and quality due to the conflict between biological and social rhythms. Insufficient sleep has been associated with worse physical health status and irregular eating behaviors in adolescents. This review aims to systematically synthesize the longitudinal associations between adolescents’ sleep dimensions (i.e., duration, timing, quality, and insomnia symptoms) and physical health indicators (i.e., anthropometric indices, fat percentage, and risk of obesity), eating behaviors, and nutritional aspects (i.e., type of diet related to the intake of specific foods and nutrients, amount and timing of food consumption, energy expenditure). A total of 28 longitudinal studies were included. The meta-analytic results showed that longer sleep duration, better sleep quality, and lower insomnia symptoms were associated with lower BMI and fat percentage and that shorter sleep duration (<7 h) and lower sleep quality were associated with a higher risk of obesity. Conversely, anthropometric indices were not related to sleep over time. Limited literature examined the bidirectional association between adolescents’ sleep and their eating behaviors and nutritional aspects. Such knowledge sheds new light on the role of sleep for adolescents’ health, highlighting the need to examine further the interplay between these variables.

## 1. Introduction

**Figure 1:**
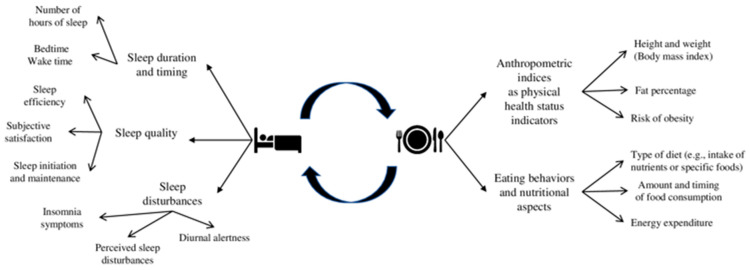
Overview of the main dimensions.

Sleep is a fundamental human psychophysiological function, and good sleep quality is essential for adolescents’ healthy development [1]. Sleep quality can be conceived as a multi-faceted phenomenon referring to satisfaction with sleep, adequate sleep duration, regular sleep timing, initiation and maintenance of sleep, and the ability to maintain alertness during waking hours [2] (see Figure 1 for a summary of the main dimensions of sleep considered). According to the National Sleep Foundation [3], adolescents (aged 14–17 years) are recommended to sleep around 8–10 h and to maintain a regular sleep timing (keeping almost the same bedtimes and wake-up times amongst weekdays and weekends). These recommendations are considered central assets for promoting adolescents’ physical health (e.g., weight control, hormones regulation) [3,4,5]. However, over the last decades, adolescents’ sleep duration has been significantly reduced, becoming a source of public health concern [6,7]. This dramatic decline could be due to the interplay between biological (e.g., changes in melatonin secretion; for a review, [8]) and psychosocial changes (e.g., irregular sleep patterns and changes in physical activity and digital use; for reviews, [9,10]) that happen during adolescence.

Importantly, poor sleep quality can be associated with adolescents’ main indicators of physical health status (i.e., weight status, risk of developing obesity), as well as with their eating behaviors and nutritional aspects (i.e., type of diet related to the intake of nutrients or specific foods, amount and timing of food consumption and energy expenditure) [11,12,13] (see Figure 1 for a summary of the main dimensions considered). Nowadays, there is a trend of increasing obesity rates among adolescents [14] and that could lead to a greater risk of non-communicable diseases such as cardiovascular diseases and type 2 diabetes [15], as well as psychosocial problems later in life, such as depression [16] and bullying [17]. Moreover, eating behaviors and nutritional aspects become more irregular during adolescence in terms of type of diet, amount and timing of food consumption (e.g., skipping breakfast and decline in fruit and vegetable consumption), and energy expenditure [18,19,20]. Moving from this knowledge of an interplay between sleep, physical health status indicators, eating behaviors, and nutritional aspects [11,12,13], this systematic review with meta-analysis aims to shed light on the available longitudinal findings of how adolescents’ sleep, anthropometric indices, eating behaviors, and nutritional aspects are bidirectionally related over time.

### 1.1. The Interplay between Adolescents’ Sleep and Anthropometric Indices

Anthropometric indices are crucial indicators of the health status of individuals. For example, waist circumference, body fat percentage, and Body Mass Index (BMI, weight/height^2^; kg/m^2^) are some of the most used measures of body fatness in children and adolescents [21]. Moreover, these indicators are helpful in identifying several pathologies. Indeed, based on the World Health Organization, adolescents’ overweight and obesity are defined as a gender- and age-specific BMI at or above the 95th percentile on the growth references [22]. Over the past decades, adolescents’ obesity has become a global public health issue since its association with cardiometabolic and psychosocial comorbidity [23,24].

Previous results highlighted an association between short sleep duration (less than the amount of sleep recommended for the considered age group), increased BMI, and obesity among adolescents (for reviews, [12,25,26,27,28]). One of the mechanisms that was hypothesized to be involved in this relation is the biological alteration in appetite-regulating hormones ghrelin (which stimulates appetite) and leptin (which restrains appetite). Specifically, short sleep duration has been shown to increase ghrelin [29] and decrease leptin [30]. However, results about the effects of sleep on the secretion of leptin and ghrelin are still mixed (for a review [31]), also suggesting that longer sleep duration can be associated with lower levels of leptin [32] or higher levels of ghrelin [33]. Most of these results are based on cross-sectional studies that included both children and adolescents, so the inconsistency may be partially explained by participants’ heterogeneity (e.g., age, sex, and weight status) and studies’ methodological differences (e.g., measurement tools and participant selection) [30]. 

Another possible explanation of the link between poor sleep quality, short sleep duration, and higher BMI and consequent risk of obesity could be attributed to the trend of adolescents to prefer a pattern of behavior referred to as eveningness, characterized by later bedtime and wake-up times and an increase in activity levels later on the day [34,35,36,37,38]. In addition, adolescents’ preference for eveningness contrasts with their social schedules (i.e., school entrance time) and often results in short sleep duration [39,40,41]. This can also have diurnal consequences such as daytime sleepiness and higher sedentary time or fatigue, leading to reduced energy expenditure and, consequently, a predisposition to obesity [42], creating a vicious circle. 

These findings highlight the potential role adolescents’ poor sleep can play in negatively affecting their weight status, predisposing them to developing obesity. Conversely, less is known about the opposite direction of this relation. Thus, among the adult population, only a few studies showed that obesity could be an important risk factor for daytime sleepiness and nocturnal sleep disturbances (for a review [43]), while among adolescents, evidence is even rarer. In conclusion, both poor sleep and obesity are increasing among adolescents, with major consequences for their health. Nevertheless, there is a lack of knowledge on the mechanism and sleep related aspects involved in this reciprocal association.

### 1.2. The Interplay between Adolescents’ Sleep, Eating Behaviors, and Nutritional Aspects

In adolescence, youth tend to modify their eating habits, becoming more irregular in timing and diet pattern. Specifically, they tend to drink more energy-dense foods and sweetened beverages, eat away from home more often (e.g., in fast food restaurants), and increase portion sizes [44,45,46]. Dietary patterns formed during adolescence tend to be maintained even in adulthood [47,48,49]. Among the adult population, studies found a bidirectional link between sleeping and eating: on the one hand, sleep duration could influence hunger (e.g., [50]) and emotional eating (e.g., eating in response to aroused emotional states, [51]) and, on the other hand, some kind of food such as ones that contain melatonin and tryptophan (e.g., fruits and vegetables) and high-carbohydrate diets could be associated with sleep quality and quantity [52,53]. 

Compared to the adult population, less attention has been paid to the interplay between adolescents’ eating behavior and sleep over time. Previous cross-sectional and laboratory studies focused on the role of sleep duration, showing that adolescents’ sleep deprivation (assessed with both objective and subjective measures) led to higher caloric intake and changes in food choices (e.g., greater consumption of high-sugar snacks and dessert) [54,55,56]. Moreover, sleep timing was found to be related to eating patterns (e.g., later bedtimes were associated with higher intake of energy-dense food [56,57,58]). Therefore, sleep timing could be associated with meal timing and, doing so, weight status (i.e., individuals with later bedtimes who wake up later in the morning may skip breakfast and engage in sedentary activity, such as using digital media, during the evening [58,59,60].

Considering the reverse relation between eating pattern and sleep, previous studies found that adolescents with a tendency to consume greater amounts of highly processed food (e.g., sweets and candy, baked goods, pastries), skip breakfast, and have irregular eating schedules tend to show poorer sleep quality, higher sleep-related problems (e.g., insomnia), and less restorative sleep [61,62,63,64,65]. Additionally, dietary patterns considered healthy, such as the Mediterranean diet (e.g., high consumption of plant-based foods; moderate intake of dairy products, seafood, eggs, and poultry; olive oil as the primary fat source; and low consumption of red meat [66,67]) has been shown to be associated with longer adolescents’ sleep duration and lower odds of reporting sleep-related problems [68,69]. Nevertheless, studies on this direction of the association are limited and provide only a scattered picture of this phenomenon.

### 1.3. The Present Study 

Considering the literature previously examined, on the one hand, shorter sleep duration could be related to higher BMI or more irregular eating behaviors (in terms of timing and type of food consumption) (e.g., [54,56]). On the other hand, eating behaviors characterized by regular timing and healthy nutritional patterns (e.g., high consumption of plant-based food) could be associated with longer sleep duration and less sleep-related problems among adolescents [68,69]. However, some knowledge gaps need to be addressed in the available literature. 

First, most of the studies used cross-sectional designs, making it impossible to examine the different directions of the relation (e.g., [70]); second, they included different age groups without differentiating between children and adolescents (e.g., [71]); third, they mainly focused on the effect of sleep on physical health variables and mostly considered a single aspect of sleep (e.g., sleep duration [72]), not providing a comprehensive understanding of the phenomenon. Similarly, concerning eating behaviors variables, previous studies mainly focused on the negative side of dietary patterns, disregarding how the type of diet or the ordinary amount of food intake are associated with sleep over time. Because of these criticisms, prior systematic reviews and meta-analyses (e.g., [12]) could provide only a partial picture of the complex longitudinal interplay between adolescents’ sleep, eating behaviors, and nutritional aspects.

The current systematic review with meta-analysis sought to address these gaps by reviewing longitudinal studies focusing on adolescents’ samples and considering how different aspects of sleep and adolescents’ physical health status indicators, potentially related eating behaviors and nutritional aspects, are bidirectionally linked. This can provide a more comprehensive picture of how this reciprocal relation develops over time, clarifying its direction and the different implications of the variables considered. In this vein, this study aims to systematically summarize previous longitudinal evidence on the bidirectional link between sleep variables (i.e., sleep duration, timing, quality, and insomnia symptoms) and (a) anthropometric indices and obesity risk and (b) eating behaviors and nutritional aspects (i.e., type of diet, amount and timing of food consumption, and energy expenditure) (an overview of the factors examined is presented in Figure 1).

## 2. Materials and Method

This review conformed to the Preferred Reporting Items for Systematic Reviews and Meta-Analyses (PRISMA) guidelines [73] (see Document S1 for the PRISMA checklist, PROSPERO preregistration ID: CRD42021281002). The current study is part of a larger project aimed to review available longitudinal research evaluating the interplay between sleep and different aspects of psychosocial development in adolescents, e.g., [74,75,76].

### 2.1. Eligibility Criteria

Specific eligibility criteria were defined regarding the studies and publications’ characteristics. To meet eligibility criteria, studies needed to (a) include adolescents from the general population as a sample (attending junior or secondary high schools and aged between 10 and 19 years old); (b) use a longitudinal study design (with at least two assessments); (c) examine at least one aspect of sleep measured with either objective (e.g., actigraphy, polysomnography) and subjective measures (e.g., questionnaires, sleep diaries, clinical interviews); (d) examine at least one variable related to anthropometric indices as physical health status indicators (i.e., BMI, fat percentage, and risk of obesity) and/or related eating behaviors and nutritional aspects (i.e., type of diet related to the intake of specific foods and nutrients, amount and timing of food consumption, and energy expenditure) (for details, see Figure 1). Moreover, concerning the publication’s characteristics, both peer-reviewed journal articles and grey literature that can be retrieved through database searches (e.g., doctoral dissertations) were included to avoid selection biases and reinforce the methodological rigor [77]. Finally, no restrictions were applied based on the year and the language of publication (when articles/dissertations were published in a language other than English, professional translators were contacted).

### 2.2. Literature Search

The literature search was conducted on the 28th of January 2023 to detect research published in peer-reviewed journal articles or available as grey literature. The full list of databases searched with full query strings used for each are reported in the Supplemental materials, Documents S2. Moreover, using the statistics of the previous search conducted in Web of Science, the websites of the fifteen journals that published most on the topic were screened (for the full list, see Document S3). Afterward, sleep-related conference proceedings (i.e., *Journal of Sleep Research and Sleep Medicine*) and the reference lists of the most relevant published systematic reviews and meta-analyses (e.g., [78]) (see Document S4 for the full list) were screened. The last step consisted of screening the reference lists of included studies. The searches and the screening were run and managed on Citavi 6 software.

### 2.3. Selection of Studies

**Figure 2:**
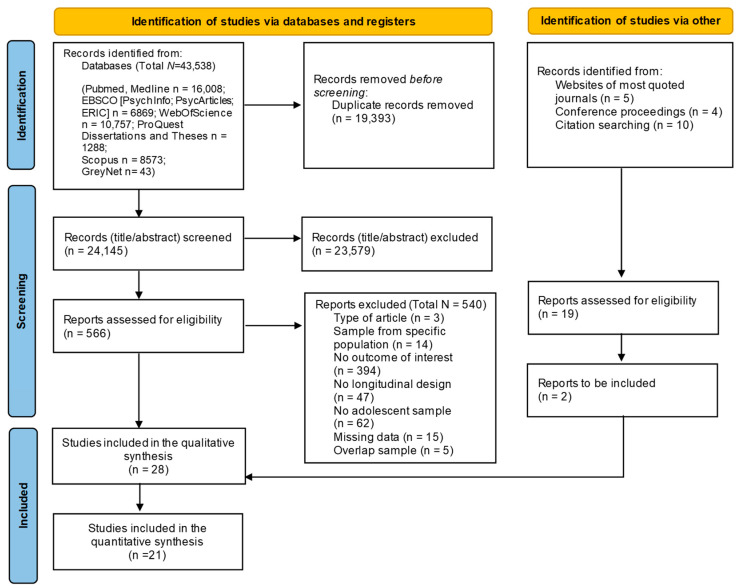
PRISMA diagram search flow.

The results of the search strategies are reported in the PRISMA diagram (Figure 2). The searches yielded 43,538 abstracts, and after removing duplicates, two raters independently screened the remaining records (*N* = 24,145) with a substantial percentage of agreement (Cohen’s Kappa = 0.77), and disagreements were discussed with a third rater (taking the final decisions with the achievement of the agreement among all raters). Next, the full texts were screened following the same procedure used for the abstract screening (the agreement was high; Cohen’s Kappa = 0.63). In total, 28 studies were included in the systematic review, with 21 also included in the meta-analytic calculation.

### 2.4. Coding of Primary Studies

All the included studies were coded independently and simultaneously by two independent raters (V.B. and M.G.) (the percentage of agreement was 91.8%) following a coding protocol. The disagreements were discussed with a third rater and solved among the three evaluators. In the first part of the protocol, the publications’ characteristics were coded: type of publication (i.e., journal article or grey literature), year of publication, and the publication language. Next, studies’ characteristics were grouped: funding sources; the number of waves of the longitudinal design; the interval between waves; the dimensions of each study; and the assessment method categorized as subjective (e.g., questionnaires, sleep diaries, clinical interviews) and objective assessment (e.g., actigraphs, polysomnography). Third, participants’ characteristics were coded: sample size, gender composition of the sample (% females), mean age, geographical location, and ethnic composition of the sample.

Finally, data necessary for effect size computations were coded. Due to the high heterogeneity of the studies included, different effect sizes were extracted (i.e., odds ratios, risk ratios, cross-lagged correlations, beta coefficients, means and standard deviations) to address how sleep variables, anthropometric indices, eating behaviors, and nutritional aspects were longitudinally related (see Section 2.6). If only standardized beta regression coefficients were reported, the correlation coefficients were estimated based on Peterson and Brown’s formula [79]. If it was not possible to obtain data for effect size computation from the primary studies, authors were contacted by e-mail to request them. Specifically, a total of 26 authors were contacted; if they did not answer, one reminder (after two weeks) was scheduled. Seven authors replied by providing the requested data; seven replied that they could not provide data (e.g., they did not have the access to the data); and 12 did not respond. The total number of studies included in the review accounts for 15 that were excluded because of insufficient data, as indicated in the PRISMA diagram (Figure 2).

### 2.5. Methodological Quality Assessment and Risk of Bias

In line with the PRISMA guidelines [73], each study’s methodology quality and risk of bias were evaluated. The quality assessment of studies was performed by the first two authors (the percentage of agreement based on the 20% of the included studies was 83%) by using an adapted version of the Newcastle–Ottawa Scale (NOS) for cohort studies following previous procedures (e.g., [74]). Specifically, the adapted version of the NOS included six items categorized into three dimensions: Selection, Comparability, and Outcome. A series of response options are provided for each item, and a star system is used, assigning a maximum of one star for each Selection and Outcome item and a maximum of two stars for the Comparability item to high-quality studies. Document S5 of the Supplemental Materials shows the full list of items.

### 2.6. Strategy of Analysis

Data related to sleep variables measured at one time point (e.g., sleep duration at T1) and anthropometric indices, eating behaviors, and nutritional aspect variables at the last time-point considered in the study (T2), or anthropometric indices, eating behaviors, and nutritional aspects variables at one time point (e.g., BMI at T1) and sleep at the last time-point considered in the study (T2) were coded. Concerning the bidirectional relation between sleep, anthropometric indices (i.e., BMI and fat%), eating behavior, and nutritional aspect variables, the effect sizes were converted into Pearson’s correlations to compare the effects across studies and compute overall summary statistics through meta-analytic techniques. Pearson’s correlations were converted into Fisher’s Z-scores for computational purposes and converted back into correlations for presentation [80]. Correlations of |.10| were considered small, |.30| moderate, and |.50| large effect sizes [81,82]. Moreover, to estimate the quantitative relation between sleep and risk for obesity, the estimated odds ratio from each study comparing the shortest sleep duration and lowest sleep quality to the normative sleep duration and the highest level of sleep quality on the risk of obesity were used. 

The computation of variance, standard error, 95% confidence interval, and statistical significance were applied for each effect size. When at least three studies [83,84] were available on the same association, a meta-analysis was conducted using the software ProMeta3 to obtain an overall estimate. The random-effect model was used as a conservative approach to account for different sources of variation among studies (i.e., within-study and between-studies variance; [85]).

Furthermore, in order to assess heterogeneity through included studies the *Q* statistic (to test the significance) and the I2 (for the estimation, considering 25% as low, 50% as moderate, and 75% as high proportion of dispersion) were used [86]. Moderator analyses were performed to examine which factors can account for the heterogeneity [87] when at least three studies for each moderator level were available [84]. Specifically, subgroup analysis (for categorical moderators, i.e., method used to assess sleep) and meta-regression (for numerical moderators, i.e., age of participants and time-lag between waves). Finally, the evaluation of the publication bias was performed through the visualization of the funnel plot (i.e., a scatter plot of the effect sizes estimated from individual studies against a measure of their precision, such as their standard errors). When the plot appears as a symmetrical inverted funnel, it indicates no presence of bias. Nevertheless, since smaller or non-significant studies are less likely to be published, studies in the bottom left-hand corner of the plot are frequently absent. Together with the visualization of the funnel plot, in order to test the statistical asymmetry of the funnel plot, Egger’s regression method [88] was used (with non-significant results indicating the absence of publication bias).

## 3. Results

### 3.1. Study Characteristics and Quality Assessment

Twenty-eight studies were included in the systematic review. A summary of the characteristics of the included studies is reported in Table 1. For what concerns the publications’ characteristics, most of the studies were articles published in peer-reviewed journals (*n* = 27, 96.4%), and only one [89] was a dissertation. All studies were published in English, and approximately half of them (*n* = 15, 53.5%) were published recently between 2019 and 2023, with the remaining published before 2019. Concerning the study design, one study [90] was a daily actigraphic study with 7 days of assessment. The remaining studies mostly used two time points (*n* = 21, 75%), with only a small percentage that used three (*n* = 2, 7.1%), or more than three (*n* = 4, 14.3%), time points. The average time lag between adjacent waves was about two years (M = 28.0 months, SD = 23.1 months), ranging from 6 months to 7 years. Most of the studies (*n* = 20, 71.4%) evaluated sleep variables through subjective measures (i.e., ad hoc questions or questionnaires), the remaining (*n* = 7, 25%) assessed sleep using objective evaluation (i.e., actigraphy), and one used both methods of assessment (*n* = 1, 3.6%). Moreover, regarding the considered sleep variables, most of the studies (*n*= 17, 61%) focused only on the sleep duration dimension, seven studies evaluated sleep timing, seven evaluated sleep quality variables, and only one evaluated insomnia symptoms. For what concerns the anthropometric indices, eating behaviors, and nutritional aspect variables, most of the studies focused only on the BMI assessment (*n* = 17), three focused also on food intake, six on the type of diet, and one on the energy expenditure. Most studies (*n* = 25, 89.3%) reported one or multiple funding sources. The total number of participants was 118,291 (M = 3960, SD = 5823, range 59–20,745). Most samples were gender balanced (the average percentage of females across samples was 51.6%; range 42.6–65.3%), and the average age of sample participants at baseline was 13.4 years (SD = 2.5, range: 8.1–18 years). With regards to the geographic context of the studies, most of them were conducted in the USA (*n* = 11, 39.3%). The remaining studies were conducted in Europe (*n* = 7, 25%), Australia (*n* = 3, 10.7%), China (*n* = 3, 10.7%), Canada (*n* = 2, 7.1%), Brazil (*n* = 1, 3.6%), and Mexico (*n* = 1, 3.6%). Results of the methodological quality and risk of bias assessment are reported in Document S6 of the Supplemental Materials. The overall quality of the studies was high, with a consequent low risk of bias.

### 3.2. Longitudinal Interplay between Adolescents’ Sleep, Anthropometric Indices, Eating Behaviors, and Nutritional Aspects

In Table 2, every effect size of the included studies is reported. Twenty studies reported the needed effect sizes for the meta-analytic calculations. Results of the meta-analytic calculations for the longitudinal interplay between sleep variables, anthropometric indices, obesity risk, eating behaviors, and nutritional aspect variables are reported below.

#### 3.2.1. The Interplay between Sleep, Anthropometric Indices, and Risk of Obesity

Regarding the interplay between sleep and anthropometric indices (i.e., BMI, fat%), 18 studies examined this link either unidirectionally or bidirectionally. Regarding the longitudinal association between sleep variables at one time point (T1) and anthropometric indices at a later time (T2), 15 studies [89,91,92,94,97,98,100,103,107,108,110,112,115,116,123] were included in the meta-analytic results, while three studies [117,120,122] reported only qualitative information. Results of the meta-analysis, summarized in Table 3 (See Document S7 for the forest plot), showed that the overall effect size was significant, albeit small (*r* = −0.06, *p* < 0.001). Specifically, longer sleep duration, higher sleep quality, and lower presence of sleep disturbances (i.e., insomnia symptoms) were related to lower BMI and fat percentage in adolescents over time. Heterogeneity statistics were high and significant. Nevertheless, the results were not moderated by the characteristics of the participants (i.e., mean age at T1, *B* = 0.00, *p* = 0.76), by characteristics of the studies (i.e., time-lag between waves, *B* = 0.00, *p* = 0.98), or by sleep assessment method (*Q* = 0.29, *p* = 0.59). The visual investigation of the funnel plot suggested a risk for publication bias, supported by the results of the Egger’s test. Results of the studies not included in the meta-analysis confirmed this association [117] when considering sleep duration but not when considering the sleep trajectories over time as predictors [120,122]. 

Examining the opposite direction, seven studies [89,91,92,97,108,115,116] focused on the association between anthropometric indices at one point (T1) and sleep variables at a later time (T2), which allowed for a meta-analytical calculation. As shown in Table 3 (see Document S8 for the forest plot), anthropometric indices were not significantly related to sleep quality and duration over time. This result was not moderated by participants’ age at T1 (*B* = −0.00, *p* = 0.89) or by the time-lag between waves (*B* = −0.00, *p* = 0.38), and was not affected by publication bias, as evident from the non-significant Egger’s test (*p* = 0.18).

Regarding the interplay between sleep and the risk of obesity over time, seven studies [95,96,114,118,119,121,124] examined this link either unidirectionally or bidirectionally. Regarding the longitudinal association between sleep variables at one time point (T1) and obesity risk at a later time (T2), six studies [95,96,118,119,121,124] were included in the meta-analytic results, while one study [114] reported only qualitative information. As shown in Table 3 (see Document S9 for the forest plot), the meta-analytic result was significant but small (OR: 1.30 [1.08,1.56], *p* < 0.01). Specifically, shorter sleep duration (<7 h) and lower sleep quality were associated with higher risk of obesity in adolescents over time. This result was not moderated by participants’ age at T1 (*B* = 0.02, *p* = 0.75) or by the time-lag between waves (*B* = −0.01, *p* = 0.06), and was not affected by publication bias, as evident from the non-significant Egger’s test (*p* = 0.11). Results of the study not included in the meta-analysis confirmed these findings [114]. Finally, only one study [118] evaluated this connection bidirectionally, showing no significant association between obesity at one time point and short sleep duration at a later time point. 

#### 3.2.2. The Interplay between Sleep, Eating Behaviors, and Nutritional Aspects 

Four studies [90,101,104,108] evaluated the link between sleep, different aspects of eating behaviors, and nutritional aspects (i.e., type of diet related to the number of specific foods and nutrients, amount and timing of food consumption, and energy expenditure). Given their heterogeneity in considering different eating behavior and nutritional aspect indicators, only a qualitative review of the findings was conducted. The main findings are summarized in Table 2. Regarding the association between sleep variables at one time point and eating behaviors and nutritional aspects at a later time point, one study [104] showed that longer sleep duration was associated with junk food consumption over time. This result was not confirmed by the other study that evaluated this link [101], which highlighted no association between sleep quality and healthy dietary quality as assessed by the Healthy Eating Index 2015 [102] over time. Moreover, one study evaluated the association between sleep duration and caloric intake over time, showing no significant association [108]. Finally, regarding the relation between sleep and energy expenditure over time, only one study [90] found a negative association between sleep duration with energy expenditure over time. Examining the opposite direction, only one study [104] evaluated the association between junk food consumption at one time point and sleep at a later time point. Results showed a significant association between unhealthier eating behaviors and longer sleep duration over time.

## 4. Discussion

Nowadays, an alarming decline in adolescents’ sleep duration and disruption of their sleep quality and healthy habits was highlighted [7,126], and at the same time, adolescents’ obesity has become a global public health issue [22]. However, there is a lack of awareness of the possible bidirectional link between these variables over time. For these reasons, the present systematic review with meta-analysis aimed to improve the knowledge of the interplay between youth’ sleep on the one hand and physical health status indicators, eating behaviors, and nutritional aspects on the other by systematically evaluating longitudinal studies specifically focused on adolescents’ samples. Overall, the findings of this study highlighted composite association patterns between sleep and physical health variables in adolescents. Such knowledge sheds new light on the role of sleep as an essential component of health, especially during adolescence, and shows up new directions for future research examining how healthy eating behaviors and good sleep quality could bidirectionally influence each other.

### 4.1. Is Sleeping Well Longitudinally Related to Anthropometric Indices?

Meta-analytic results showed a small and negative association between different aspects of sleep (i.e., sleep duration, timing, quality, and insomnia symptoms) and anthropometric indices over time. Specifically, longer sleep duration, higher sleep quality, and lower presence of sleep disturbances (i.e., insomnia symptoms) were related to lower BMI and fat percentage in adolescents over time, assessed with subjective and objective measures. Despite being small, this effect aligns with prior cross-sectional research (for reviews, see [25,27]). Moreover, this result is partially in line with previous cross-sectional studies among the adult population in which longer sleep duration was also associated with higher BMI [127,128,129].

On the contrary, only a few studies investigated the other direction of this link, mainly considering BMI levels and subsequent sleep duration. The reviewed findings highlighted the absence of a significant association between these factors, as higher BMI was not associated with lower sleep duration and quality. Nevertheless, it is important to underline the potentially crucial role that BMI might play in the link between poor sleep and the risk of obesity. Specifically, on the one hand, poor sleep quality can be associated with a higher level of BMI, and on the other hand, higher levels of BMI and fat percentage in adolescents can be associated with lower levels of physical activity, leading to an impairment in physical health that can, in turn, be related to youth’ sleep, suggesting the establishment of a possible vicious circle. For this reason, more research is needed considering several aspects of adolescents’ physical health to understand the longitudinal interplay between sleep and anthropometric indices.

Additionally, considering the association between adolescents’ sleep and the risk of obesity, meta-analytic results showed that short sleep duration and poor sleep quality had a small but significant effect on the future risk of obesity over time. This result reflects previous cross-sectional evidence suggesting a possible link between poor sleep quality and overweight/obesity in young subjects (for a review, see [12]). Moreover, this aligns with previous studies among the adult population (for reviews, see [130,131]. This result can be explained through several mechanisms. First, short-term sleep duration has been shown to increase daytime sleepiness and related sedentary behaviors, which can, in turn, reduce daytime physical activity [132]. Second, adolescents’ biological phase delay is misaligned with their early school start time, leading to the implementation of compensative behavior for their sleep debt during the weekends. One of these typical behaviors is the delay of bedtime and wake-up time during weekends compared to weekdays, known as social jet lag [41]. This phenomenon was found to be strongly related to an increased risk of developing obesity and metabolic syndrome (e.g., [133]). Third, sleep deprivation can be related to increased energy expenditure that, in turn, results in compensatory behaviors such as increased food intake [134]. For these reasons, it is of utmost importance to consider all these mechanisms when evaluating the relation between sleep and obesity risk over time to provide a comprehensive understanding of sleep’s potential role during development.

The small effect found should be read in light of the fact that most of the included studies considered sleep duration as a predictor of a high risk of obesity (considering “short sleep” category as a duration of around 6–7 h, compared to the “normal” sleep duration category for adolescents of 8–10 h, as suggested by the National Sleep Foundation guidelines). Since, as stated above, adolescents are normally sleep deprived, this comparison could not be so effective in predicting the risk of obesity over time for this specific age group. Conversely, other sleep dimensions can be crucial in detecting this risk. Moreover, the average time-lag between the assessments was approximately two years so several factors might have interceded. Future studies could benefit from including multiple evaluations of sleep, considering additional more qualitative aspects of sleep when evaluating this relation (i.e., sleep latency and maintenance), and using multiple time points of assessment in order to clarify the role of time in this association.

### 4.2. Is Sleeping Well Longitudinally Related to Eating Behaviors and Nutritional Aspects?

The results of this systematic review suggest that there is not a clear picture of the bidirectional association between adolescents’ sleep, their eating behavior, and nutritional aspects. This is in line with previous findings [50], highlighting a mixed picture of the relation between sleep quality, eating behaviors, and nutritional aspects over time. Of the included studies, only four examined this link, but considering different indicators; therefore, only a qualitative review of the findings could be conducted. Most of the studies evaluated the association between sleep at one time point and eating behaviors and nutritional aspects at a later time, and only one explored this association bidirectionally, highlighting a significant positive association between longer sleep duration and junk food consumption [104]. One study [108] evaluated how sleep duration and caloric intake were related over time, showing no significant associations. Moreover, one study [90] assessed the association between sleep quality and energy expenditure, finding a negative association over time. 

Considering the limited literature on the longitudinal association between different aspects of sleep, eating behaviors, and nutritional aspects in adolescents, there is a need to deepen the knowledge of this interplay. On the one hand, previous cross-sectional studies highlighted the importance of considering a wider framework of sleep factors (e.g., sleep regularity) associated with eating behaviors (e.g., [135]) and not only sleep duration. On the other hand, previous evidence mainly focused on the pathological expression of eating behaviors (e.g., [136]) on youths’ general adjustment, but less is known about the role of a non-disordered eating on sleep. 

### 4.3. Limitations and Future Research Directions

The results of this systematic review with meta-analysis are not free from limitations. The first one concerns the factthat despite sleep being a multidimensional factor, most of the included studies measured it as a unidimensional variable, mostly considering sleep duration as an indicator of good sleep quality. Future studies need to uncover the relative impact of each aspect considering sleep’s multidimensionality (considering both healthy and pathological aspects and typical behaviors that adolescents implement to compensate for their sleep debt). Moreover, most of the examined studies evaluated the link between sleep and physical health in a unidirectional way. Only eight studies (e.g., [118]) considered the bidirectionality of the relation. Therefore, future prospective studies are needed to elucidate how the different facets of sleep are related to different physical health variables over time and vice versa. 

Second, most studies included employed self-report measures (e.g., [104,112], specifically, only seven used actigraphy to assess sleep objectively (e.g., [97,101]), and only nine used objective methods to assess anthropometric indices (e.g., [90,103]. Thus, self-report data should be considered cautiously because they may be affected by social desirability, memory biases, and shared variance issues [137]. Future studies should integrate objective and subjective measures to assess physical health variables: along with objective information about anthropometric indices obtained from digital scaling and a stadiometer [138], day-to-day variability intake or patterns of intake frequency and timing could allow for more valid data [139]. Similarly, it is of utmost importance to evaluate sleep parameters through actigraphy in future studies [140] in order to provide a reliable and more nuanced picture of healthy adolescents’ sleep compared to self-report, which might overestimate sleep duration [141]. Moreover, a greater consistency between studies regarding methodology and assessment methods is required in order to better compare them in terms of sample considered, assessment method, statistical analyses, and study design.

Third, most included studies used two time points in their study designs. Future studies should evaluate the longitudinal link between sleep and physical health in adolescents with at least three assessments to better understand how (i.e., underlying mechanisms) they are related over time and for whom (i.e., moderations) this association is stronger. In this way, it would be possible to identify relevant mediators (e.g., physical activity) playing a role in the interplay between sleep quality and physical health variables in adolescents (e.g., higher BMI → higher sedentariness → shorter sleep duration, [142]). Furthermore, it is crucial to clarify the short-, medium-, and long-term nature of these effects. This understanding is of great importance to developing evidence-based interventions.

Finally, the included studies mainly focused on the interplay between adolescents’ sleep and physical health status indices. This highlights a gap in the literature on the specific role of eating behaviors and nutritional aspects in relation to adolescents’ sleep. Future studies should focus more on the multidimensionality of human eating behaviors and nutrition variables, considering the combined role of anthropometric indices and other aspects of eating behaviors and nutrition, such as the intake of specific foods and time of food consumption.

## 5. Conclusions

This is the first systematic review with a meta-analysis that provided a comprehensive synthesis of the literature regarding the longitudinal research on the association between sleep, physical health status indicators, eating behaviors, and nutritional aspects in adolescents. First, meta-analytic results revealed a weak but significant association between sleep at one time point and anthropometric indices and obesity risk at a later time. Moreover, meta-analytic results in the opposite direction showed a non-significant effect. These results suggest that future studies should clarify the existence of a vicious circle between sleep and physical health variables, also exploring their effect on sleep, considering all the dimensions. 

From a theoretical perspective, this review calls attention to some gaps in the present literature. To address them, future studies should highlight the importance of considering the bidirectionality of the relation between adolescents’ sleep quality and physical health, conducting longitudinal studies that examine both qualitative and quantitative aspects of all the considered variables. Likewise, this study has important practical implications. Considering the bidirectionality of this relation, it will be possible to implement evidence-based interventions aimed at promoting well-being in adolescence by educating about sleep hygiene rules and practices to improve one’s sleep health and encouraging healthier eating patterns.

## Figures and Tables

**Table 1:** Characteristics of studies included in the systematic review.

Study	Characteristics of the Studies						Characteristics of the Participants						
Authors and Year	Funding	*N* Waves	Time Lag	Sleep Dimension (Method of Assessment)	Sleep Assessment	Anthropometric Indices, Eating Behaviors, and Nutritional Aspects Variables (Method of Assessment)	Anthropometric Indices, Eating Behaviors, and Nutritional Aspect Variables Assessment	*N* Participants Baseline	*N* Participants Follow-Up	% Females	Mean Age (in Years) at Baseline	Country	% Ethnicity
Ames et al., 2016 [91]	National funding	6	2 years	Sleep duration (S)	Ad hoc question: hours slept per night	BMI (S)	Self report	662	477	51.7%	15.5	Canada	Caucasian: 85.0%
Araujo et al., 2012 [92]	National funding	2	5 years	Sleep duration (S)	Ad hoc questions: usual bedtimes and wake-up times on weekdays	BMI, type of diet (intake of specific foods and nutrients) (S)	Self report; Food Frequency Questionnaire (FFQ) [93]	2160	1171	n/a	n/a	Portugal	n/a
Bagley et al., 2015 [94]	National funding	2	1 year	Sleep duration, sleep quality, sleep timing (O)	Actigraphs	BMI (O)	Stadiometer and digital weight scale	274	256	44.1%	10.4	USA	European American: 64.0%; African American: 36.0%
Calamaro et al., 2010 [95]	National funding	2	1 year	Sleep duration (S)	Ad hoc question: hours slept per night	BMI, Type of diet (intake of specific foods and nutrients) (S)	Self reported unhealthy eating patterns	20,745	13,568	49.7%	16	USA	White, non-Hispanic: 67.1%; Black, non-Hispanic: 15.4%; Hispanic: 12.3%; Other 5.3%
Cao et al., 2018 [96]	National funding	2	9 months	Sleep duration (S)	Ad hoc question: hours slept per night	BMI, Type of diet (intake of specific foods and nutrients) (S)	Ad hoc question about the daily intake of meat, sugar beverages, fruits, and vegetables	18,302	14,089	52.9%	11.3	China	n/a
Chong et al., 2021 [97]	Multiple funding	2	6 months	Sleep duration (O)	Actigraphs	BMI (O)	Stadiometer and digital scale	127	88	57.5%	11.1	Australia	n/a
Collings et al., 2015 [98]	National funding	2	2.5 years	Sleep duration (S)	Sleep Habits Survey for Adolescents [99]	BMI (O)	Stadiometer and digital scale	504	504	57.7%	15.0	UK	White: 92.8%
Danielsen et al., 2021 [100]	National funding	2	6 years	Sleep duration (S)	Ad hoc questions: bedtimes and wake-up times	BMI (S)	Self report	5781	3025	54.9%	11.7	Norway	n/a
Fairborn, 2010 [89]	National funding	2	1 year	Sleep duration, sleep quality (S)	Ad hoc questions: hours of sleep per night and whether they usually get enough sleep	BMI (S)	Self report	14,723	14,723	51.0%	n/a	USA	White: 51.1%; African American: 21.5%; Hispanic: 16.5%; Asian: 6.8%; American Indian: 2.6%; no ethnic identity: 1.5%
Full et al., 2021 [101]	National funding	3	1 year	Sleep duration, sleep quality, sleep timing (O)	Actigraphs	Type of diet (intake of specific foods and nutrients) (S)	Healthy Eating Index 2015 [102]	455	423	46.5%	15.2	USA	White: 87.0%; Non-white: 13.0%
Fung et al., 2022 [103]	Multiple funding	2	2 years	Sleep duration (S)	Ad hoc questions: hours slept per night	BMI (O)	By measuring weight and height	10,574	9273	48.0%	9.9	USA	African American: 14.8%; American Indian and Alaska Native: 0.5%; Asian: 2.2%; Pacific Islander: 0.1%; White: 66.0%; Other: 4.0%; Two or more races: 12.3%
Gardner et al., 2022 [104]	Multiple funding	2	1 year	Sleep duration (S)	Modified Sleep Habits Survey [105]	Food intake (S)	Items adapted from the NSW School Physical Activity and Nutrition Survey [106]	983	983	54.8%	12.6	Australia	n/a
Gong et al., 2020 [107]	Multiple funding	2	2 years	Sleep duration (S)	Ad hoc questions: bedtimes and wake-up times	BMI, (intake of specific foods and nutrients) (S)	Ad hoc questionnaire	1901	1510	49.2%	12.2	China	n/a
Jansen et al., 2020 [108]	Multiple funding	2	2 years	Sleep duration, sleep timing (S+O)	Sleep diary + actigraphs	BMI (O), Type of diet (intake of specific foods and nutrients) (S), food intake (S)	Weight and height; Food Frequency Questionnaire (FFQ) [109]	554	458	54.0%	14.4	Mexico	Mexican: 100%
Jindal et al., 2020 [90]	n/a	Daily	7 days	Sleep duration, sleep quality, sleep timing (O)	Actiheart	BMI, energy expenditure (O)	Stadiometer and digital balance; doubly labelled water (DWL) method	59	59	n/a	8.1	USA	49.1% Hispanic; 27.1% Black; 22.0% White; 1.7% Asian
Kracht et al., 2023 [110]	Multiple funding	2	2 years	Sleep duration (O)	Accelerometry	BMI (O), (intake of specific foods and nutrients) (S)	A trained study staff member measured weight and height; Automated Self-Administered 24-Hour Dietary Assessment Tool (ASA-24 2016 [111])	286	217	51.8%	12.9	USA	White: 58.1%; African American: 36.4%; Other ethnicity: 5.5%
Lang et al., 2019 [112]	National funding	2	10 months	Insomnia Symptoms (S)	Insomnia Severity Index [113]	BMI (S)	Self report weight and height	1242	864	42.6%	18	Switzerland	n/a
Lim et al., 2019 [114]	Multiple funding	2	6 years	Sleep duration, sleep timing (S)	Ad hoc questionnaire	BMI (S)	Self report	580	516	62.4%	12.8	China	n/a
Maume, 2017 [115]	National funding	2	4 years	Sleep duration (S)	Ad hoc questions: usual bedtimes and wake-up times	BMI (S)	Self report	974	974	50.0%	n/a	USA	Non-White: 19.0%
Merikanto et al., 2020 [116]	Multiple fundings	2	5 years	Sleep duration, sleep quality, sleep timing (O)	Actigraphs	BMI (S)	Measured at the clinical visit	353	167	53%	12.3	Finland	n/a
Mitchell et al., 2013 [117]	National funding	8	6 months	Sleep duration (S)	Self reported typical duration of sleep on a school night	BMI (S)	Self reported weight and height	1336	1089	50.0%	14.3	USA	Black: 14.0%; White: 74.6%; Other: 11.5%
Roberts et al., 2015 [118]	National funding	2	1 year	Sleep duration (S)	Ad hoc questions on presence, frequency, and duration of insomnia symptoms	BMI (S)	Self report	4175	3134	49.2%	n/a	USA	European American: 37.0%; African American: 34.6%; Latino American: 23.6%; Other: 4.7%
Saelee et al., 2020 [119]	National funding	2	1 year	Sleep duration (S)	Ad hoc question: hours slept per night	BMI (S)	Self report	12,692	12,692	49.6%	15.0	USA	White: 66.0%; African American: 14.8%; Asian: 12.2%; Hispanic: 3.9%; Other: 3.1%
Schafer et al., 2016 [120]	No funding	2	7 years	Sleep duration (S)	Ad hoc questions: bedtimes and wake-up times	BMI (O)	Stadiometer and digital scale	3974	3974	50.4%	n/a	Brazil	n/a
Seegers et al., 2021 [121]	Multiple funding	4	1 year	Sleep duration (S)	Ad hoc questions: bedtimes and wake-up times	BMI (S)	Self report	3017	1916	47.2%	n/a	Canada	n/a
de Souza et al., 2015 [122]	n/a	4	1 year	Sleep duration (S)	Ad hoc questions: hours slept per night	BMI, type of diet (intake of specific foods and nutrients) (S)	Self report; ad hoc questionnaire	2245	959	48.7%	n/a	Portugal	n/a
Stefansdottir et al., 2020 [123]	Multiple funding	2	2 years	Sleep duration, sleep quality, sleep timing (O)	Actigraphs	BMI (S)	Self report	276	145	65.3%	15.9	Iceland	n/a
Wake et al., 2010 [124]	Multiple funding	3	T1-T2: 3 years; T2-T3: 5 years	Sleep quality (S)	One item from the Pittsburgh Sleep Quality Index [125] about sleep quality	BMI (O), type of diet (intake of specific foods and nutrients) (S)	Stadiometer and digital scale; eight items from the Adolescent Dieting Scale	1943	923	49.4%	16.0	Australia	n/a

Note: all included studies were journal articles, except for Fairborn 2010 [89], which was a doctoral dissertation. Abbreviations: S, subjective assessment; O, objective assessment.

**Table 2:** Effect sizes of the included studies.

Study	Sleep Variable (Method of Assessment)	Anthropometric Indices, Eating Behaviors, and Nutritional Aspects Variable (Method of Assessment)	Main Effects Reported in the Study of Sleep Variables T1 → Anthropometric Indices, Eating Behavior, and Nutritional Aspect Variables T2 ^1^	Sleep Variables T1 → Anthropometric Indices, Eating Behavior, and Nutritional Aspect Variables T2 ^1^ (Computed Effect Size Expressed as Pearson’s Correlations or Odds Ratio)	Main Effects Reported in the Study of Anthropometric Indices, Eating Behavior and Nutritional Aspect Variables T1 → Sleep Variables T2 ^2^	Main Effects Reported in the Study of Anthropometric Indices, Eating Behavior, and Nutritional Aspect Variables T1 → Sleep Variables T2 ^2^	Main Findings
Ames et al., 2016 * [91]	Sleep duration (S)	BMI (S)	*r* = −0.05	*r* = −0.05 [−0.13, 0.03]	*r* = −0.04	*r* = −0.04 [−0.12, 0.04]	No bidirectional association was found between BMI and sleep duration over time.
Araujo et al., 2012 * [92]	Sleep duration (S)	BMI (S)	Females: *r* = 0.01 Males: *r* = −0.10 *	Females: *r* = 0.01 [−0.07, 0.08] Males: *r* = −0.10 * [−0.18, −0.01]	Females: *r* = 0.00 Males: −0.09 *	Females: *r* = 0.00 [−0.08, 0.08] Males: *r* = −0.09 * [−0.17, −0.01]	Longer sleep duration was negatively and bidirectionally associated with BMI over time in males but not in females. The association was small.
Bagley et al., 2015 * [94]	Sleep duration (O)	BMI (O)	*r* = −0.19 **	*r* = −0.19 ** [−0.31, −0.07]			Longer sleep duration was associated negatively with BMI over time. The association was small. No association was found for sleep efficiency.
	Sleep quality (O)		*r* = −0.04	*r* = −0.04 [−0.16, 0.08]			
Calamaro et al., 2010 * [95]	Sleep duration (S)	Obesity risk (S)	<6 h (reference 8 to <11) OR: 1.57 [0.94, 2.62]	OR: 1.57 [0.94, 2.62]			No association was found between short sleep duration of less than 6 h and a higher risk for obesity over time.
Cao et al., 2018 * [96]	Sleep duration (S)	Obesity risk (S)	Younger males (12–15 years) <7 h: 17/450 9–11 h: 34/1411 Younger females (11–14 years) <7 h 12/366 9–11 h: 44/1595 Older males (15–17 years) <7 h: 14/405 7–9 h: 12/391 Older females (16–17 years) <7 h: 30/970 7–9 h: 32/882	Younger males (12–15 years) OR: 1.59 [0.88, 2.87] Younger females (11–14 years) OR: 1.19 [0.62, 2.29] Older males (15–17 years) OR: 1.13 [0.52, 2.48] Older females (16–17 years) OR: 0.85 [0.51, 1.41]			No association was found between short sleep duration of less than 7 h and a higher risk for obesity over time.
Chong et al., 2021 * [97]	Sleep duration (O)	BMI (O)	*r* = −0.18	*r* = −0.18 [−0.36, −0.00]	*r* = −0.12	*r* = −0.12 [−0.31, 0.07]	No bidirectional association was found between BMI and sleep duration over time.
Collings et al., 2015 * [98]	Sleep duration (S)	Fat% (O)	Males: *β* = −0.13 * (−0.27, −0.00) Females: *β* = 0.05 (−0.15, 0.25)	*r* = −0.13 [−0.26, 0.01]			Longer sleep duration was negatively associated with fat% over time in males but not in females. The association was small.
				*r* = 0.10 [−0.02, 0.21]			
Danielsen et al., 2021 * [100]	Sleep duration (S)	BMI (S)	*β* = −0.04 *	*r* = −0.04 * [−0.07, −0.00]			Longer sleep duration was negatively associated with BMI over time. The association was small.
Fairborn, 2010 * [89]	Sleep duration (S)	BMI (S)	*r* = −0.03 ***	*r* = −0.03 *** [−0.05, −0.01]	*r* = −0.02	*r* = −0.02 * [−0.04, −0.00]	Longer sleep duration was bidirectionally and negatively associated with BMI over time. The association was small. No association was found between sleep quality and BMI.
	Sleep quality (S)		*r* = 0.01	*r* = 0.01 [−0.01, 0.03]	*r* = 0.00	*r* = 0.00 [−0.02, 0.02]	
Full et al., 2021 [101]	Sleep duration (O)	Healthy eating habits (S)	*β* = −0.58 (0.88)	*r* = 0.52 [−0.59, −0.45]			No longitudinal associations were found between sleep characteristics and overall dietary quality over time.
	Sleep quality (O)		*β* = 0.38 (0.44)	*r* = 0.37 [0.29, 0.45]			
	Midpoint of sleep (O)		*β* = 0.43 (0.45)	*r* = 0.42 [−0.50, −0.34]			
Fung et al., 2022 * [103]	Sleep duration (S)	BMI (O)	BMI T1 Adolescents with adequate sleep duration at T1: Mean (*SD*): 17.90 (3.27) Adolescents without adequate sleep duration at T1: Mean (*SD*): 19.04 (3.86) BMI T2: Adolescents with adequate sleep duration at T1: Mean (*SD*): 19.57 (*3.96*) Adolescents without adequate sleep duration at T1: Mean (*SD*): 21.00 (*4.56*)	*r* = −0.03 ** [−0.06, −0.01]			Adolescents without adequate sleep duration at baseline (9–11 h of sleep) showed a higher increase in BMI compared to adolescents with adequate sleep duration at baseline. The association was small.
Gardner et al., 2022 [104]	Sleep duration (S)	Unhealthy eating (S)	*r* = 0.22 **	*r* = 0.22 ** [0.16, 0.28]	*r* = 0.14 **	*r* = 0.14 ** [0.08, 0.20]	Longer sleep duration was associated positively and bidirectionally with junk food consumption over time.
Gong et al., 2020 * [107]	Sleep duration (S)	BMI (S)	*r* = −0.10 ***	*r* = −0.10 *** [−0.15, −0.05]			Longer sleep duration was negatively associated with BMI over time. The association was small.
Jansen et al., 2020 * [108]	Sleep duration (O)	BMI (O)	*r* = −0.09	*r* = −0.09 [−0.18, 0.00]	*r* = −0.06	*r* = −0.06 [−0.15, 0.03]	Sleep duration was not associated with BMI over time and vice versa. Moreover, sleep duration was not associated with caloric intake over time.
		Caloric intake (S)	*r* = −0.01		*r* = −0.01		
Jindal et al., 2020 [90]	Sleep duration (O)	Energy expenditure (O)	*β* = −0.41 **	*r* = −0.35 ** [−0.58, −0.12]			Sleep duration was negatively associated with energy expenditure over time.
Kracht et al., 2023 * [110]	Sleep duration (O)	Fat% (O)	*β* = −0.00	*r* = −0.00 [−0.13, 0.13]			No association was found between sleep duration and fat% over time.
Lang et al., 2019 * [112]	Sleep disturbances (S)	BMI (S)	*β* = 0.06 *	*r* = −0.11 ** [−0.17, −0.04]			Higher sleep disturbances were associated with higher BMI over time. The association was small. No association was found between sleep quality and BMI over time.
	Sleep quality (S)		*β* = −0.04	*r* = −0.04 [−0.11, 0.03]			
Lim et al., 2019 [114]	Sleep duration (S)	Obesity risk (S)	RR: 1.40 [0.54, 3.67]				Sleep duration was not associated with a higher risk of obesity over time.
Maume, 2017 * [115]	Sleep duration (S)	BMI (S)	*β* = −0.21 *	*r* = −0.21 *** [−0.27, −0.15]	*β* = 0.01	*r* = 0.01 [−0.05, 0.07]	Longer sleep duration was associated with lower BMI over time, but not vice versa. This association was small.
Merikanto et al., 2020 * [116]	Sleep duration (O)	BMI (O)	*r* = −0.10 *	*r* = −0.10 [−0.25, 0.05]	*r* = −0.06	*r* = −0.06 [−0.21, 0.09]	Longer sleep duration was associated with lower BMI over time, but not vice versa. This association was small. No association was found between midpoint of sleep, wake after sleep onset, and BMI over time.
	Midpoint of sleep (O)		*r* = −0.08	*r* = 0.08 [−0.07, 0.23]	*r* = −0.02	*r* = 0.02 [−0.13, 0.17]	
	Wake after sleep onset (O)		*r* = −0.02	*r* = 0.02 [−0.13, 0.17]	*r* = −0.07	*r* = 0.07 [−0.08, 0.22]	
Mitchell et al., 2013 [117]	Sleep duration (S)	BMI (O)	n/a	n/a	n/a	n/a	Each additional hour of sleep was associated with a reduction in BMI over time.
Roberts et al., 2015 * [118]	Sleep duration (S)	Obesity risk (S)	<6 h OR: 1.29 [0.90–1.84]	<6 h OR: 1.29 [0.90–1.84]	<6 h OR: 1.04 [0.76–1.42]	<6 h OR: 1.04 [0.76–1.42]	No association was found between short sleep duration (less than 6 h) and the risk of obesity over time.
Saelee et al., 2020 * [119]	Sleep duration (S)	Obesity risk (S)	*r* = 0.11 *	OR: 1.49 *** [1.40–1.59]			Short sleep duration was associated with a higher risk of obesity over time. The association was small
Schafer et al., 2016 [120]	Sleep duration (S)	BMI, fat% (O)	n/a	n/a	n/a	n/a	Girls who increased their sleep duration from 11 to 18 years of age showed an increase in BMI and fat mass index.
Seegers et al., 2021 * [121]	Sleep duration (S)	Obesity risk (S)	Short Sleepers vs. 11 h OR: 1.24 [0.38–2.09]	Short Sleepers vs. 11 h: OR: 1.24 [0.38–2.09]			Sleep duration was not associated with a higher risk of obesity over time.
de Souza et al., 2015 [122]	Sleep duration (S)	BMI (S)	n/a	n/a	n/a	n/a	Sleep duration was not associated with BMI trajectories over time.
Stefansdottir et al., 2020 * [123]	Sleep duration (O)	BMI (S)	*β* = −0.04	*r* = −0.04 [−0.20, 0.13]			Sleep duration was not associated with BMI over time.
Wake et al., 2010 * [124]	Sleep quality (S)	Obesity risk (O)	Poor sleep quality vs. good sleep quality: OR: 0.79 [0.46–1.36]	Poor sleep quality vs. good sleep quality: OR: 0.79 [0.46–1.36]			Sleep quality was not associated with a higher risk of obesity over time.

Notes. ^1^ Cross-lagged effects between sleep measured at one time point and anthropometric indices, eating behavior, and nutritional aspect variables measured at the last time point considered in the study. ^2^ Cross-lagged effects between anthropometric indices and eating behavior variables measured at one time point and sleep measured the last time point considered in the study. (S) = subjective assessment of sleep parameters; (O) = objective assessment of sleep parameters; M = mean and standard deviation in parentheses; β = standardized regression coefficient and standard error estimate in parentheses; r = Pearson’s correlation (confidence intervals are reported between square brackets); OR = odds ratio and confidence interval in square brackets; RR = risk ratio and confidence intervals in square brackets. *** *p* < 0.001, ** *p* < 0.01, * *p* < 0.05. Studies marked with an asterisk (*) are those included in the meta-analyses reported in Table 3.

**Table 3:** Overall meta-analytic calculations of the bidirectional relations between sleep and physical health status indicators.

Overall Effect	*k*	ES [95% CI]	*Q*	I^2^	Egger’s Test	Overall Effect	*k*	ES [95% CI]	*Q*	I^2^	Egger’s Test
Between Sleep T1 → Physical Health Variables T2 ^1^						Between Physical Health Variables T1 → Sleep T2 ^2^					
Sleep T1 → Anthropometric indices T2 ^a^	15	*r* = −0.06 ***	57.08 **	75.47	−2.40 *	Anthropometric indices T1 à Sleep T2	7	*r* = −0.01	4.35	0	−1.56
		[−0.09, −0.03]						[−0.03, −0.00]			
Poor sleep T1 → Risk of obesity T2 ^b^	6	OR = 1.30 **	8.85	43.51	−2.06						
		[1.08, 1.56]									

Notes. ^1^ Cross-lagged effects between sleep measured at one time point (T1) and physical health status indicators measured at the last time point (T2) considered in the study. ^2^ Cross-lagged effects between physical health status indicators measured at one time point (T1) and sleep measured at the last time point (T2) considered in the study. *k* = number of studies; ES = effect Size; *Q* = heterogeneity test; I^2^ = heterogeneity estimate. *** *p* < 0.001, ** *p* < 0.01, * *p* < 0.05. ^a^ To compute the overall meta-analytic summary, the effect sizes of studies were recoded so that higher sleep quality, longer sleep duration, and lower levels of sleep disturbances at T1 were related to higher anthropometric measures (i.e., BMI, fat%) at T2. ^b^ To compute the overall meta-analytic summary, the effect sizes of studies were recoded so that shortest sleep duration and lowest sleep quality (compared to the normative sleep duration and the highest level of sleep quality) at T1 were related to higher risk for obesity at T2.

## Data Availability

Data from previously published studies were retrieved and analyzed. Data sharing is not applicable to this article.

## Supplementary Materials

The following supporting information can be downloaded at https://www.mdpi.com/article/10.3390/nu15143179/s1. Document S1: PRISMA checklist. Document S2: Full search strategy for each database. Document S3: Full list of screened journals. Document S4: Full list of most relevant published systematic reviews and meta-analyses. Document S5: Quality and Risk of Bias Assessment Method (adapted from the Newcastle-Ottawa Scale for Cohort Studies). Document S6: Risk of Bias Assessment Results. Document S7: Forest plot for the association between sleep variables at one time point (T1) and anthropometric indices at a later time (T2). Document S8: Forest plot for the association between anthropometric indices at one point (T1) and sleep variables at a later time (T2). Document S9: Forest plot for the association between sleep variables at one time point (T1) and obesity risk at a later time (T2). References [27,28,78,143,144,145,146,147,148,149,150,151,152,153] are cited in supplementary file.
