# Supplementary material for: The Longitudinal Interplay between Sleep, Anthropometric Indices, Eating Behaviors, and Nutritional Aspects: A Systematic Review and Meta-Analysis
Source: Nutrients. 2023 Jul 18;15(14):3179. doi: 10.3390/nu15143179 (PMC10385596; doi:10.3390/nu15143179)

Supplemental Materials

Document S1: PRISMA Checklist

Section and Topic	Item #	Checklist item	Location where item is reported
**TITLE**			
Title	1	Identify the report as a systematic review.	Title Page
**ABSTRACT**			
Abstract	2	See the PRISMA 2020 for Abstracts checklist.	Page 1
**INTRODUCTION**			
Rationale	3	Describe the rationale for the review in the context of existing knowledge.	Pages 1-4
Objectives	4	Provide an explicit statement of the objective(s) or question(s) the review addresses.	Page 4
**METHODS**			
Eligibility criteria	5	Specify the inclusion and exclusion criteria for the review and how studies were grouped for the syntheses.	Pages 4, 5
Information sources	6	Specify all databases, registers, websites, organisations, reference lists and other sources searched or consulted to identify studies. Specify the date when each source was last searched or consulted.	Page 5
Search strategy	7	Present the full search strategies for all databases, registers and websites, including any filters and limits used.	Page 5
Selection process	8	Specify the methods used to decide whether a study met the inclusion criteria of the review, including how many reviewers screened each record and each report retrieved, whether they worked independently, and if applicable, details of automation tools used in the process.	Page 5
Data collection process	9	Specify the methods used to collect data from reports, including how many reviewers collected data from each report, whether they worked independently, any processes for obtaining or confirming data from study investigators, and if applicable, details of automation tools used in the process.	Page 6
Data items	10a	List and define all outcomes for which data were sought. Specify whether all results that were compatible with each outcome domain in each study were sought (e.g. for all measures, time points, analyses), and if not, the methods used to decide which results to collect.	Page 6
	10b	List and define all other variables for which data were sought (e.g. participant and intervention characteristics, funding sources). Describe any assumptions made about any missing or unclear information.	Page 6
Study risk of bias assessment	11	Specify the methods used to assess risk of bias in the included studies, including details of the tool(s) used, how many reviewers assessed each study and whether they worked independently, and if applicable, details of automation tools used in the process.	Page 6
Effect measures	12	Specify for each outcome the effect measure(s) (e.g. risk ratio, mean difference) used in the synthesis or presentation of results.	Pages 6, 7
Synthesis methods	13a	Describe the processes used to decide which studies were eligible for each synthesis (e.g. tabulating the study intervention characteristics and comparing against the planned groups for each synthesis (item #5)).	Pages 6, 7

Section and Topic	Item #	Checklist item	Location where item is reported
	13b	Describe any methods required to prepare the data for presentation or synthesis, such as handling of missing summary statistics, or data conversions.	Pages 6, 7
	13c	Describe any methods used to tabulate or visually display results of individual studies and syntheses.	Pages 6, 7
	13d	Describe any methods used to synthesize results and provide a rationale for the choice(s). If meta-analysis was performed, describe the model(s), method(s) to identify the presence and extent of statistical heterogeneity, and software package(s) used.	Pages 6, 7
	13e	Describe any methods used to explore possible causes of heterogeneity among study results (e.g. subgroup analysis, meta-regression).	Pages 6, 7
	13f	Describe any sensitivity analyses conducted to assess robustness of the synthesized results.	Pages 6, 7
Reporting bias assessment	14	Describe any methods used to assess risk of bias due to missing results in a synthesis (arising from reporting biases).	Page 6
Certainty assessment	15	Describe any methods used to assess certainty (or confidence) in the body of evidence for an outcome.	Page 6
**RESULTS**			
Study selection	16a	Describe the results of the search and selection process, from the number of records identified in the search to the number of studies included in the review, ideally using a flow diagram.	Page 5, Figure 1
	16b	Cite studies that might appear to meet the inclusion criteria, but which were excluded, and explain why they were excluded.	Figure 1
Study characteristics	17	Cite each included study and present its characteristics.	Pages 7-8
Risk of bias in studies	18	Present assessments of risk of bias for each included study.	Page 8, Document S6
Results of individual studies	19	For all outcomes, present, for each study: (a) summary statistics for each group (where appropriate) and (b) an effect estimate and its precision (e.g. confidence/credible interval), ideally using structured tables or plots.	Pages 8-27
Results of syntheses	20a	For each synthesis, briefly summarise the characteristics and risk of bias among contributing studies.	Page 8, Document S6
	20b	Present results of all statistical syntheses conducted. If meta-analysis was done, present for each the summary estimate and its precision (e.g. confidence/credible interval) and measures of statistical heterogeneity. If comparing groups, describe the direction of the effect.	Pages 8-27
	20c	Present results of all investigations of possible causes of heterogeneity among study results.	Pages 8-27
	20d	Present results of all sensitivity analyses conducted to assess the robustness of the synthesized results.	Pages 8-27
Reporting biases	21	Present assessments of risk of bias due to missing results (arising from reporting biases) for each synthesis assessed.	Page 8, Document S6
Certainty of evidence	22	Present assessments of certainty (or confidence) in the body of evidence for each outcome assessed.	Page 8, Document S6

Section and Topic	Item #	Checklist item	Location where item is reported
**DISCUSSION**			
Discussion	23a	Provide a general interpretation of the results in the context of other evidence.	Pages 28-30
	23b	Discuss any limitations of the evidence included in the review.	Pages 29, 30
	23c	Discuss any limitations of the review processes used.	Pages 29, 30
	23d	Discuss implications of the results for practice, policy, and future research.	Pages 29, 30
**OTHER INFORMATION**			
Registration and protocol	24a	Provide registration information for the review, including register name and registration number, or state that the review was not registered.	Page 4
	24b	Indicate where the review protocol can be accessed, or state that a protocol was not prepared.	Page 4
	24c	Describe and explain any amendments to information provided at registration or in the protocol.	n/a
Support	25	Describe sources of financial or non-financial support for the review, and the role of the funders or sponsors in the review.	31
Competing interests	26	Declare any competing interests of review authors.	31
Availability of data, code and other materials	27	Report which of the following are publicly available and where they can be found: template data collection forms; data extracted from included studies; data used for all analyses; analytic code; any other materials used in the review.	n/a

From: Page MJ, McKenzie JE, Bossuyt PM, Boutron I, Hoffmann TC, Mulrow CD, et al. The PRISMA 2020 statement: an updated guideline for reporting systematic reviews. BMJ 2021;372:n71. doi: 10.1136/bmj.n71

For more information, visit: http://www.prisma-statement.org/

Document S2: Full search strategy for each database

PubMed and MEDLINE

(((Sleep*[Title/Abstract] OR insomnia[Title/Abstract] OR polysomnogra*[Title/Abstract] OR REM[Title/Abstract] OR actigraph*[Title/Abstract] OR EEG [Title/Abstract] OR motor activity [Title/Abstract] OR circadian*[Title/Abstract] OR chronotype[Title/Abstract]) AND (pediatr*[Title/Abstract] OR paediatr*[Title/Abstract] OR teen*[Title/Abstract] OR school*[Title/Abstract] OR adolescen*[Title/Abstract] OR youth*[Title/Abstract] OR young*[Title/Abstract] OR child*[Title/Abstract])) AND (longitudinal*[Title/Abstract] OR prospective*[Title/Abstract] OR follow-up[Title/Abstract] OR daily[Title/Abstract] OR day-to-day[Title/Abstract] OR wave[Title/Abstract])))

EBSCO [PsycINFO, PsycArticles, ERIC]

AB (Sleep* OR insomnia OR polysomnogra* OR REM OR actigraph* OR EEG OR motor activity OR circadian* OR chronotype*) AND AB (pediatr* OR paediatr* OR teen* OR school* OR adolescen* OR youth* OR young* OR child*) AND AB (longitudinal* OR prospective* OR follow-up OR daily OR day-to-day OR wave)

Web of Science

AB=(Sleep* OR insomnia OR polysomnogra* OR REM OR actigraph* OR EEG OR motor activity OR circadian* OR chronotype*) AND AB=(pediatr* OR paediatr* OR teen* OR school* OR adolescen* OR youth* OR young* OR child*) AND AB=(longitudinal* OR prospective* OR follow-up OR daily OR day-to-day OR wave)

ProQuest Dissertations and Theses

ab(Sleep* OR insomnia OR polysomnogra* OR REM OR actigraph* OR EEG OR motor activity OR circadian* OR chronotype*) AND ab(pediatr* OR paediatr* OR teen* OR school* OR adolescen* OR

youth* OR young* OR child*) AND ab(longitudinal* OR prospective* OR follow-up OR daily OR day-to-day OR wave)

Scopus

(ABS (sleep* OR insomnia OR polysomnogra* OR rem OR actigraph* OR eeg OR motor AND activity OR circadian* OR chronotype*) AND ABS (pediatr* OR paediatr* OR teen* OR schoo l* OR adolescen* OR youth* OR young* OR child*) AND ABS (longitudinal* OR prospective* OR follow-up OR daily OR day-to-day OR wave))

GreyNet

(Sleep* OR insomnia OR polysomnogra* OR REM OR actigraph* OR EEG OR motor activity OR circadian* OR chronotype*) AND (pediatr* OR paediatr* OR teen* OR school* OR adolescen* OR youth* OR young* OR child*) AND (longitudinal* OR prospective* OR follow-up OR daily OR day-to-day OR wave)

Document S3: Full list of screened journals

The screened journals were (in alphabetical order):

BMC Public Health;

Brain Development;

Developmental Medicine and Child Neurology;

Epilepsia;

Epilepsy Behavior;

International Journal of Environmental Research and Public Health;

International Journal of Pediatrics Otorhinolaryntology;

Journal of Child Neurology;

Journal of Clinical Sleep Medicine;

Journal of Sleep Research;

Pediatrics;

Plos One;

Seizure European Journal of Epilepsy;

Sleep;

Sleep Medicine.

Document S4: Full list of most relevant published systematic reviews and meta-analysis

The full list of most relevant published systematic reviews and meta-analyses of which the reference lists were screened.

Beisbier, S., & Laverdure, P. (2020). Occupation-and activity-based interventions to improve performance of instrumental activities of daily living and rest and sleep for children and youth ages 5–21: A systematic review. *The American Journal of Occupational Therapy*, *74*(2), 7402180040p1-7402180040p32. https://doi.org/10.5014/ajot.2020.039636

Belmon, L. S., van Stralen, M. M., Busch, V., Harmsen, I. A., & Chinapaw, M. J. (2019). What are the determinants of children's sleep behavior? A systematic review of longitudinal studies. *Sleep medicine reviews*, *43*, 60-70. https://doi.org/10.1016/j.smrv.2018.09.007

Costa, S., Benjamin-Neelon, S. E., Winpenny, E., Phillips, V., & Adams, J. (2019). Relationship between early childhood non-parental childcare and diet, physical activity, sedentary behaviour, and sleep: A systematic review of longitudinal studies. *International journal of environmental research and public health*, *16*(23), 4652. https://doi.org/10.3390/ijerph16234652

Ehsan, Z., Ishman, S. L., Kimball, T. R., Zhang, N., Zou, Y., & Amin, R. S. (2017). Longitudinal cardiovascular outcomes of sleep disordered breathing in children: A meta-analysis and systematic review. *Sleep*, *40*(3), zsx015. https://doi.org/10.1093/sleep/zsx015

Fatima, Y., Doi, S. A. R., & Mamun, A. A. (2015). Longitudinal impact of sleep on overweight and obesity in children and adolescents: A systematic review and bias-adjusted meta-analysis. *Obesity reviews*, *16*(2), 137-149. https://doi.org/10.1111/obr.12245

Gronski, M., & Doherty, M. (2020). Interventions within the scope of occupational therapy practice to improve activities of daily living, rest, and sleep for children ages 0–5 years and their families: A

systematic review. *The American Journal of Occupational Therapy*, *74*(2), 7402180010p1-7402180010p33. https://doi.org/10.5014/ajot.2020.039545

Guo, Y., Miller, M. A., & Cappuccio, F. P. (2021). Short duration of sleep and incidence of overweight or obesity in Chinese children and adolescents: A systematic review and meta-analysis of prospective studies. *Nutrition, Metabolism and Cardiovascular Diseases*, *31*(2), 363-371. https://doi.org/10.1016/j.numecd.2020.11.001

Li, L., Zhang, S., Huang, Y., & Chen, K. (2017). Sleep duration and obesity in children: a systematic review and meta-analysis of prospective cohort studies. *Journal of paediatrics and child health*, *53*(4), 378-385. https://doi.org/10.1111/jpc.13434

Miller, M. A., Kruisbrink, M., Wallace, J., Ji, C., & Cappuccio, F. P. (2018). Sleep duration and incidence of obesity in infants, children, and adolescents: a systematic review and meta-analysis of prospective studies. *Sleep*, *41*(4), zsy018. https://doi.org/10.1093/sleep/zsy018

Miller, M. A., Kruisbrink, M., Wallace, J., O'Keeffe, A., Valint, S., Ji, C., & Cappuccio, F. P. (2017). Abstract MP090: Sleep duration predict incident obesity in childhood and adolescence: Meta-analysis of prospective studies. *Circulation*, 135(suppl_1), AMP090. https://doi/10.1161/circ.135.suppl_1.mp090

Ruan, H., Xun, P., Cai, W., He, K., & Tang, Q. (2015). Habitual sleep duration and risk of childhood obesity: Systematic review and dose-response meta-analysis of prospective cohort studies. *Scientific reports*, *5*(1), 1-14. https://doi.org/10.1038/srep16160

Scherrer, V., & Preckel, F. (2021). Circadian preference and academic achievement in school-aged students: A systematic review and a longitudinal investigation of reciprocal relations. *Chronobiology International*, *38*(8), 1195–1214. https://doi.org/10.1080/07420528.2021.1921788

Wu, Y., Gong, Q., Zou, Z., Li, H., & Zhang, X. (2017). Short sleep duration and obesity among children: A systematic review and meta-analysis of prospective studies. *Obesity research & clinical practice*, *11*(2), 140-150. https://doi.org/10.1016/j.orcp.2016.05.005

Document S5: Quality and Risk of Bias Assessment Method (Adapted from the Newcastle-Ottawa Scale for Cohort Studies)

Note: A study can be awarded a maximum of one star for each numbered item within the Selection and Outcome categories. A maximum of two stars can be given for Comparability

Selection

1) Representativeness of the sample

a) truly representative of the average adolescents in the community *
b) somewhat representative of the average adolescents in the community *
c) selected group of participants
d) no description of the derivation of the sample

2) Description of missing data patterns

a) clear description of missing data patterns and evaluation of missing completely at random (MCAR)*
b) partial description of missing data patterns*
c) no description of missing data patterns

Comparability

3) control of stability and covariates

a) study controls for stability of outcome *
b) study controls for any additional covariate *

Outcome

4) Assessment of outcome

a) objective measures *
b) Census data *
c) self-report (standardized measures) *
d) ad hoc questions
e) no description

5) Was follow-up long enough for outcomes to occur?

a) yes (provide clear rationale for the selected time lag) *
b) no

6) Attrition rate

a) complete follow up - all subjects accounted for *
b) subjects lost to follow up unlikely to introduce bias - small number lost ≥ 75 % follow up, or description provided of those lost) *
c) follow up rate < 75% (select an adequate %) and no description of those lost
d) no statement

Document S6: Risk of Bias Assessment Results

Study	Selection		Comparability	Outcome		
	Item 1	Item 2	Item 3	Item 4	Item 5	Item 6
Ames et al., 2016 [91]	*	*	**		*	c
Araujo et al., 2012 [92]	*	*	**		*	*
Bagley et al., 2015 [94]	*	*	**	*	*	*
Calamaro et al., 2010 [95]	*		*		*	
Cao et al., 2018 [96]	*	*	*		*	*
Chong et al., 2021 [97]	*	*	**	*	*	
Collings et al., 2015 [98]	*	*	*	*	*	
Danielsen et al., 2021 [100]	*		*		*	
Fairborn, 2010 [89]	*		*		*	
Full et al., 2021 [101]	*	*	**	*	*	
Fung et al., 2022 [103]	*	*	**		*	*
Gardner et al., 2022 [104]	*				*	
Gong et al., 2020 [107]	*				*	*
Jansen et al., 2020 [108]	*		**	*	*	*
Jindal et al., 2020 [90]	*		*	*	*	*
Kracht et al., 2023 [110]	*		*	*	*	*
Lang et al., 2019 [112]	*	*	*		*	
Lim et al., 2019 [114]	*	*	*		*	*
Maume, 2017 [115]	*	*	**		*	
Merikanto et al., 2020 [116]	*		*	*	*	
Mitchell et al., 2013 [117]	*		**		*	*
Roberts et al., 2015 [118]	*	*	*	*	*	*
Saelee et al., 2020 [119]	*	*	*	*	*	
Schafer et al., 2016 [120]	*		*	*	*	
Seegers et al., 2021 [121]	*	*	*		*	
de Souza et al., 2015 [122]	*		*		*	
Stefansdottir et al., 2020 [123]	*	*		*	*	*
Wake et al., 2010 [124]	*	*	*	*	*	*

Document S7: Forest plot for the association between sleep variables at one time point (T1) and anthropometric indices at a later time (T2)

	ES	95% CI
Ames et al., 2014	-0.05	-0.13 / 0.03
Araujo et al., 2012	-0.04	-0.10 / 0.01
Bagley et al., 2015	-0.12	-0.22 / -0.01
Chong et al., 2021	-0.18	-0.36 / -0.00
Collings et al., 2015	0.00	-0.08 / 0.09
Danielsen et al., 2021	-0.04	-0.07 / -0.00
Fairborn 2010	-0.01	-0.02 / 0.00
Fung et al., 2022	-0.03	-0.06 / -0.01
Gong et al., 2020	-0.10	-0.15 / -0.05
Jansen et al., 2020	-0.09	-0.18 / 0.00
Kracht et al., 2023	-0.00	-0.13 / 0.13
Lang et al., 2019	-0.07	-0.13 / -0.02
Maume, 2017	-0.21	-0.27 / -0.15
Merikanto et al., 2020	-0.00	-0.13 / 0.12
Stefansdottir et al., 2020	-0.04	-0.20 / 0.13
Overall (random-effects model)	-0.06	-0.09 / -0.03

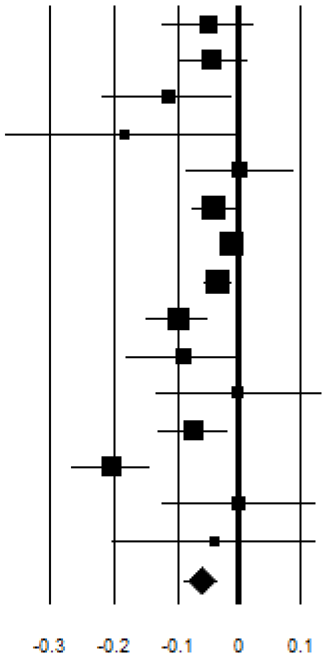

Document S8: Forest plot for the association between anthropometric indices at one point (T1) and sleep variables at a later time (T2)

	ES	95% CI
Ames et al., 2014	-0.04	-0.12 / 0.04
Araujo et al., 2012	-0.04	-0.10 / 0.02
Chong et al., 2020	-0.12	-0.31 / 0.07
Fairborn, 2010	-0.01	-0.02 / 0.00
Jansen et al., 2020	-0.06	-0.15 / 0.03
Maume, 2017	0.01	-0.05 / 0.07
Merikanto et al., 2020	0.01	-0.11 / 0.13
Overall (random-effects model)	-0.01	-0.03 / 0.00

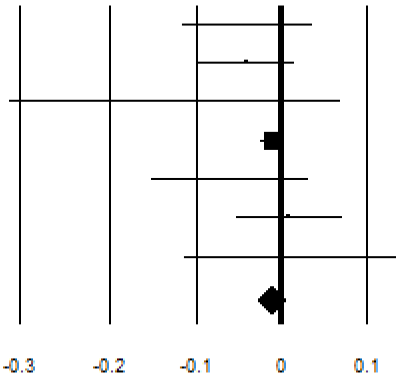

Document S9: Forest plot for the association between sleep variables at one time point (T1) and obesity risk at a later time (T2)

	ES	95% CI
Calamaro et al., 2010	1.57	0.94 / 2.62
Cao et al., 2018	1.13	0.83 / 1.53
Roberts et al., 2015	1.29	0.90 / 1.84
Saelee et al., 2020	1.49	1.40 / 1.59
Seegers et al., 2021	1.24	0.38 / 2.09
Wake et al., 2010	0.79	0.46 / 1.36
Overall (random-effects model)	1.30	1.08 / 1.56

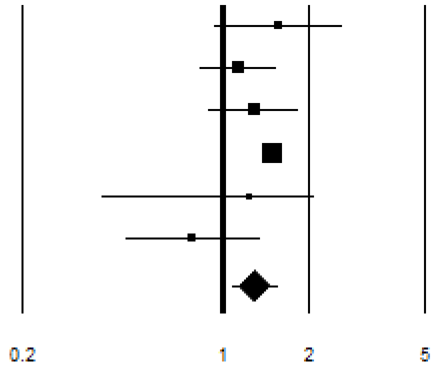

Supplement: Supplementary file 1 [file nutrients-15-03179-s001.zip › nutrients-2441881-supplementary.pdf]
